# Low temperature offers better foam stability

**DOI:** 10.1111/jocd.16398

**Published:** 2024-06-04

**Authors:** Mola Serkan, Deniz Gokay

**Affiliations:** ^1^ Department of Cardiovascular Surgery Ankara City Hospital Ankara Turkey

**Keywords:** foam, polidocanol, sclerotherapy, venous insufficiency

## Abstract

**Background:**

The foam's structural longevity, linked to the effectiveness of sclerotherapy, depends on preparation conditions. The factors enhancing the treatment's effectiveness and efficacy are still under discussion.

**Methods:**

We conducted an in vitro preclinical research, which included 144 independent trials. A total of 8 combinations involving 18 trials were designed according to settings of +4°C and room temperature (20–22°C), liquid‐to‐air ratios of 1/1 and 1/4, and polidocanol concentrations of 0.5% and 1% using the modified Tessari method. Our study aimed to examine the effect of air ratio, agent temperature, and polidocanol concentration on stability by assessing the foam half‐time (FHT) and defining the optimal preparation conditions.

**Results:**

The mean FHT was 117 ± 30.4 s. The longest FHT was in a 1:4 air‐to‐sclerosant ratio at +4°C, regardless of the sclerosant concentration (for %0.5 mean FHT: 146.2 ± 13.9 s, for % 1 mean FHT: 146.9 ± 18 s). There was a significant interaction among the three variables on FHT (*p* = 0.001). Temperature emerged as the primary factor (*F*(1, 136) = 124, *p* < 0.001, ηp^2^ = 0.477), with lower temperatures markedly enhancing the longevity (*p* < 0.001). Preparation at a temperature of 4°C resulted in an extended FHT of 32.5 s compared to 22°C (95% CI: 24.06–41.04 s).

**Conclusion:**

The temperature, agent concentration, and gas ratio significantly influence the stability of the physician‐compounded foam. The low temperature at +4°C may offer better FHT for sclerotherapy.

## 1 INTRODUCTION

Foam sclerotherapy (FS) offers a minimally invasive, cost‐effective, quick‐recovery treatment modality for reticular or telangiectasia vein treatment in chronic venous insufficiency (CVI). The procedure involves administering a sclerosant agent, which leads to inflammation and endothelial fibrosis in the targeted vein lumen. Sclerosant administration in foam form has advantages over the liquid form, including improved contact between agent and vein wall, enhanced potency, and reduced cosmetic complications such as telangiectasia matting and hyperpigmentation. Improved durability and stability impact the effectiveness of the treatment besides avoiding the adverse effects of FS.^1^ Recent preclinical trials have focused on augmenting the foam half‐time FHT.^2^, ^3^, ^4^

Polidocanol (POL), or Lauro macrogol, is a current nonionic surfactant compound. Most practitioners prefer POL with mixing air using the modified Tessari method.^5^ The technique is simple to adapt and includes a three‐way stopcock with linked two syringes, one loaded with a liquid sclerosant agent and the other with air. Even though it can be enough to reach the desired form with 20–40 quick‐mixing cycles, the structure rapidly degrades toward the liquid.^6^ The stability is closely related to the preparation settings. Trend topics in the literature contain sclerosant concentration, gas type, the liquid–air mixing ratio, agent temperature, the number of quick‐mixing cycles, injector size, the angle of the connectors, and adding surfactants.^4^ Some are not feasible for daily practice, such as mixing CO_2_, adding a surfactant, and generating with specific instruments. Easily regulating factors, such as agent temperature, concentration, and liquid–air mixing ratio, are more valid settings in outpatient clinical usage. FHT is an established method for measuring stability.^7^ Better structural form and longer FHT are related to better treatment outcomes. The preparation temperature is limited to 10°C.^3^, ^8^ However, + 4°C temperature inside the cold chain (+2 to +8 C) could be more feasible for the agent storage without freezing.

Our goal was to investigate the main and the interaction effects of the clinically relevant factors on FHT at +4°C with %0.5 and % 1 concentration of the agent and the air ratios of 1:1 and 1:4. Second, we aimed to explore the best combination for the most prolonged durability.

## 2 MATERIALS AND METHODS

The experimental combinations consisted of two agent temperature conditions (+22°C or +4°C), two agent concentration levels (0.5% or 1%), and two agent‐air ratios (1:1 or 1:4). Groups were A (+4°C; 1:1; 1%), B (+4°C; 1:4; 1%), C (+4°C; 1:1; 0.5%), D (+4°C; 1:4; 0.5%), E (+22°C; 1:1; 1%), F (+22°C; 1:4; 1%), G(+22°C; 1:1; 0.5%), and H (+22°C; 1:1; 0.5%) (referred for each combination as agent temperature; the mixing ratio of the agent and air; the agent concentration, respectively). We used Aethoxysclerol (Kreussler Pharma, Wiesbaden, Germany) containing 0.5% and 1% concentrations of POL (Lauro macrogol). We generated all trials with 1 cc agent and varied air ratios by 20‐time quick mixing through a 3‐way tap to two 5 mL syringes used only once for each independent sample using the modified Tessari method.^5^ Subsequently, we made all FHT measurements by vertically placing the syringe filled with foam and counting decay time^7^ (Figure 1). The longer the FHT, the better stability was obtained. All groups were compared in this regard. The primary outcome was to assess the factors' effects on FHT. The secondary outcome was to determine the best condition.

**FIGURE 1:**
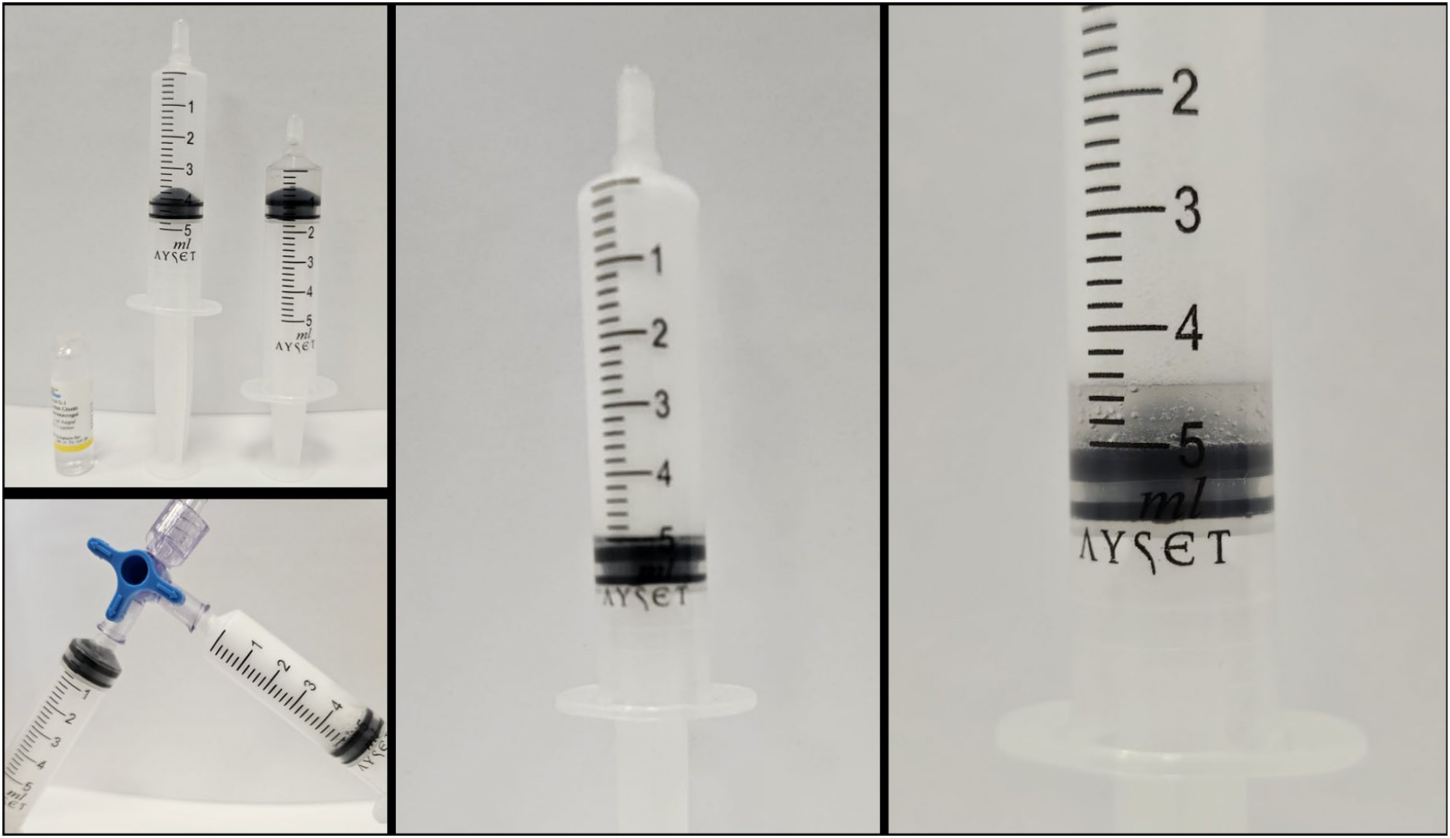
The equipment and foam‐making process. Measuring the FHT.

The power analysis with G‐Power (3.1.9.6) for three‐way ANOVA analysis indicated a total sample size of 144. We conducted eight experimental groups, each consisting of 18 independent samples. We investigated the effect of the combinations on FHT. The data for all groups included means, standard errors, and upper and lower limits for the 95% confidence intervals (95% CIs). The independent samples *t*‐test was used to compare the means of continuous variables between two different temperatures. A three‐way ANOVA test analyzes the factors' main effects and interactions. The results were in tables, including mean squares, *F* values, *p* values, and partial eta squared levels (ηp^2^). The estimated marginal means of the FHT regarding agent concentration were demonstrated. The means of groups were expressed in box plot graphics. A *p*‐value < 0.05 was considered statistically significant. All statistical analyses were performed using the SPSS for Windows version 18.0 statistical software program (SPSS Inc., Chicago, IL, USA).

## 3 RESULTS

In the study, eight groups were formed based on the combinations. We conduct a total of 144 independent trials. The overall mean time of FHT was 117 ± 30.4 s. According to the groups, the mean FHTs with the standard error are demonstrated in Figure 2. The longest FHT was in Group B and Group D (FHT: 146 ± 4.1 s and 147 ± 4.1 s, respectively). Group E had the shortest FHT (FHT: 62 ± 4.1 s 95% CI 53–70 s). Compared to the FHT according to agent temperature, the foam produced at 4°C had 32.5 s longer FHT than the foam made at room temperature (95% CI: 24.06–41.04 s).

**FIGURE 2:**
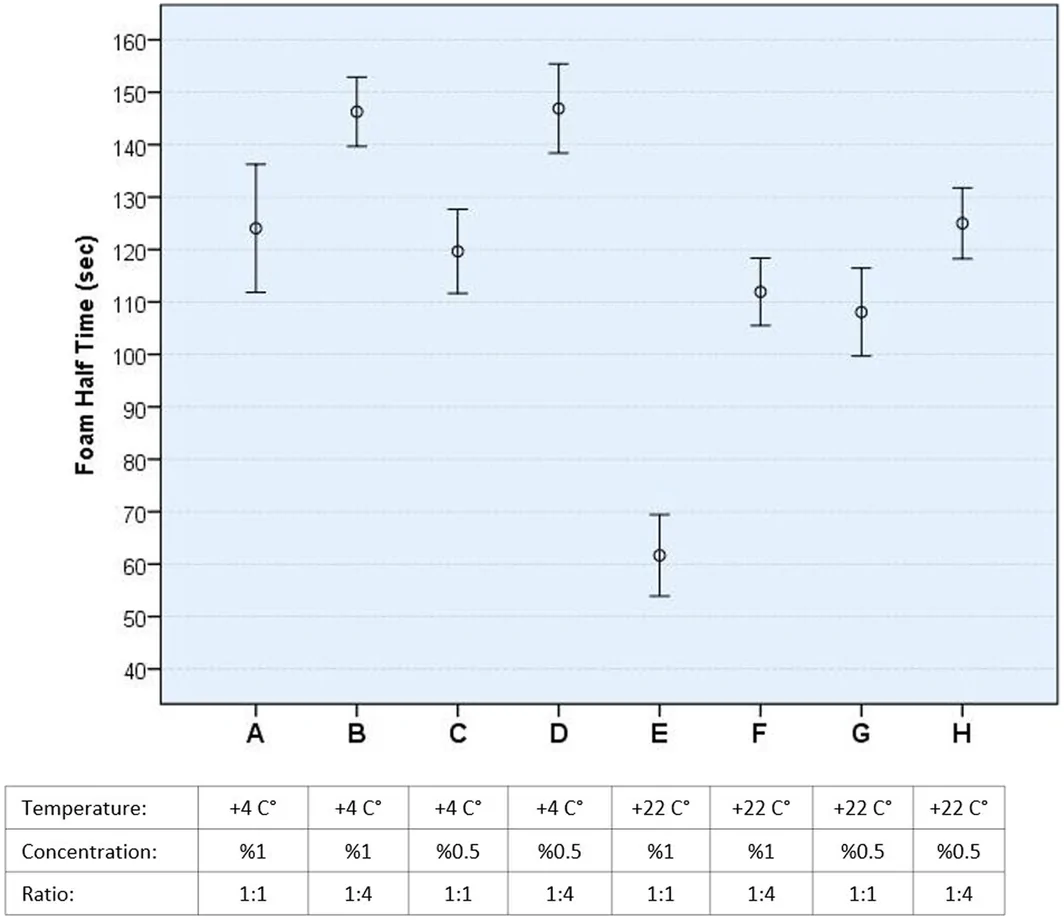
.Figure out the FHTs in groups

All three factors affected FHT. The results of multivariate analyses were documented in Table 1. The ANOVA analysis revealed the effects' importance for all settings individually (*p* < 0.001). Agent's temperature had a significant impact on FHT (*F*(1, 136) = 124, *p* < 0.001, ηp^2^ = 0.477). The ratio of air‐to‐agent mixing impacted significantly on FHT (*F*(1, 136) = 99.53, *p* < 0.001, ηp^2^ = 0.423). The concentration had the lowest main effect on FHT (*F*(1, 136) = 22.66, *p* < 0.001, ηp^2^ = 0.143). The estimated marginal FHT means of the factors were expressed in Figure 3 according to different concentrations. The interaction effect between temperature and concentration had a significant impact on FHT (*F*(1, 136) = 29.23, *p* < 0.001, ηp^2^ = 0.177) as like concentration and ratio interaction was significant (*F*(1, 136) = 5.86, *p* = 0.017, ηp^2^ = 0.041). The interaction effect between temperature and ratio was insignificant (*F*(1, 136) = 2.31, *p* = 0.131, ηp^2^ = 0.017). The three‐way interaction (temperature, concentration, and ratio) was also significant (*F*(1, 136) = 10.74, *p* = 0.001, ηp^2^ = 0.73) (Table 2). Furthermore, the temperature and the ratio of liquid agents had a more pronounced impact on FHT. Significantly, the lowest temperature of the agent mainly influenced the FHTs.

**TABLE 1:** Different measured FHTs in groups.

Temperature	Concentration	Ratio	Groups	Mean	Std. error	95% CI	
						Lower bound	Upper bound
+4 C	1%	1:1	A	124	4,1	116	132
		1:4	B	146	4,1	138	154
	0.5%	1:1	C	120	4,1	111	128
		1:4	D	147	4,1	139	155
+22 C	1%	1:1	E	62	4,1	53	70
		1:4	F	112	4,1	104	120
	0.5%	1:1	G	108	4,1	100	116
		1:4	H	125	4,1	117	133

**FIGURE 3:**
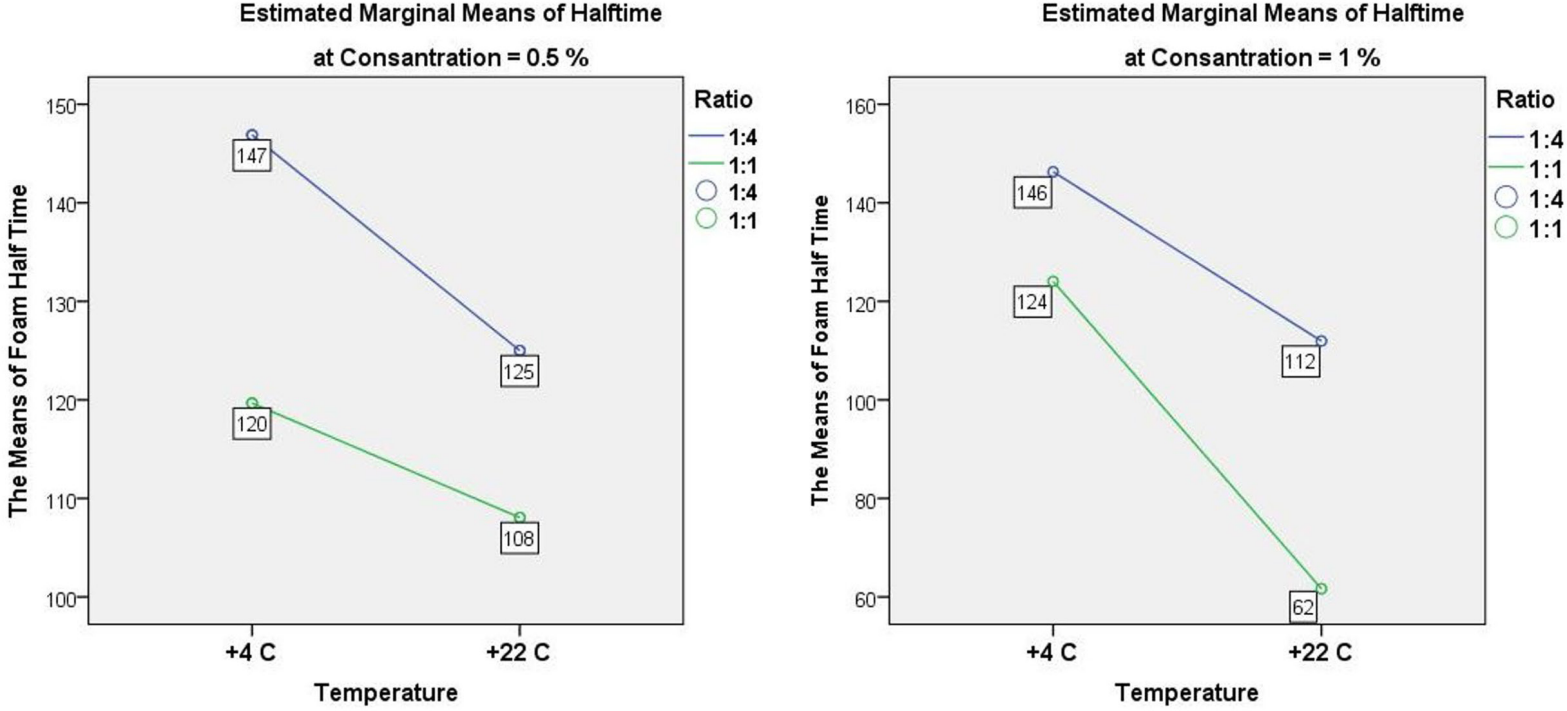
Comparing the means of FHT at +4°C and +22°C for 0.5% and 1% of agent concentration.

**TABLE 2:** The results of three‐way ANOVA for assessing the factors' effect on FHT; *R* ^2^ = 0.684 (Adjusted *R* ^2^ = 0.668).

Source	Sd	Mean square	*F*	Sig. (*p*)	Partial Eta Squared (ηp^2^)
Temperature	1	38 155	124	.000	.477
Concentration	1	6972	22.66	.000	.143
Ratio	1	30 625	99.53	.000	.423
Temperature * Concentration	1	8993	29.23	.000	.177
Temperature * Ratio	1	711	2.31	.131	.017
Concentration * Ratio	1	1806	5.87	.017	.041
Temperature * Concentration * Ratio	1	3306	10.74	.001	.073
Error	136	308			

## 4 DISCUSSION

Practitioners have widely adopted FS using the Tessari method to treat varicose veins.^9^ The technique is simple, easy, and feasible in any physician's office or clinic without any specific instrument required. Two syringes, one containing air and the other filling agent, with a three‐way tap between, are enough for physical‐compounded foam preparation by quickly mixing. Foam formation begins from the first pass. Undesirably, the form is unstable and prone to reforming back to the liquid easily. The early foam decay before applying the target vein may result in procedural complications such as matting, hyperpigmentation, and phlebitis.^10^, ^11^ However, the stability of the physician‐compounded foam may exhibit variability and depend on experience.^12^ Assessing the quality can vary due to the foam being non‐Newtonian fluid. An optimal sclerosant should possess enough cohesiveness, viscosity, and homogenous bubble sizes to demonstrate stable properties. Microstructural evaluation, assessing the range of bubble diameter, and FHT are tools for determining the foam quality.^7^ Current research in FS involves exploring various parameters on the stability, including the preparation procedure, the agent's concentration, viscosity, the air‐to‐agent preparation rate, and temperature. Experts still debate the optimal conditions for preparation. We constituted the study to facilitate straightforward implantation by practitioners in outpatient clinics and ensure reproductivity. In our results, the longest FHT was in a 0.5% drug concentration at +4°C by mixing a 1:4 ratio of air–drug. Cold temperature at +4°C improved the stability more than the mixing ratio and drug concentration.

Depending on the target vessel's diameter, practitioners prefer various agent concentrations in daily practice, such as 0.5%, 1%, 2%, and 3%. There is no clear evidence of the difference in rates of complications with different concentrations of POL.^13^ Nonetheless, the aim is to serve the optimal vessel wall destruction with the minimum attention of sclerosant as necessary for a clinical result. A 0.5% concentration should be ideal for leg telangiectasis and spiders and a %1 for varicose veins.^14^, ^15^ Our study revealed that the agent concentration could significantly affect FHT. However, whether 1% or 0.5% there was similar FHT at +4°C. Practitioners may prefer any concentration without worrying about decreased stability.

The liquid–gas ratio is another critical factor that determines the stability. Foam is a dispersion of gas bubbles in a liquid matrix, and its behavior and utilization depend on the ratio of these two phases. Bubble diameter influences gas diffusion, dryness, wetness, and inflow. As the gas‐to‐liquid ratio rises, the bubble diameter augments and fills with more air. Big bubbles are prone to collapse by having high surface tension. High agent content aids more stability since the liquid films surrounding gas bubbles are thicker, lowering the possibility of coalescing and collapsing. Likewise, higher liquid content increases viscosity, slowing drainage, gas diffusion, and stabilizing the structural form. However, above the critical sclerosant amount, adding more sclerosant can decrease the FHT due to aggravated micellization. The quadratic regression on FHT with critical micelle concentration (CMC) achieves the top at an uncertain amount. Current research has indicated this point as 1:3, 1:4, or 1:5.^4^ More than 1:4 liquid‐to‐air ratio coarsened FHT by exacerbating the fusion of the bubbles.^16^ In our study, a ratio of 1:4 liquid–air had higher FHT rather than 1:1.

Temperature significantly impacts the stability, affecting both the formation and longevity. There are some effects, such as changes in surface tension, alterations in the viscosity of the agent, changes in the solubility of gas, and the transition of foam to liquid phase. The previous research revealed that foam stability improved with lower temperatures.^3^, ^8^, ^16^, ^17^ However, the lower limit in temperature is not clear. We found that the agent temperature was the most crucial factor and the primary safety component of the foam stability. Preparing foam at +4°C significantly extended the duration of FHT regardless of the mixing ratio or concentration. +4°C as cold chamber temperature with ease to maintain in hospital or outpatient clinics offers practitioners more durable foam. Foam stability may be improved by using an agent at +4 C. Furthermore, applying cold foam can facilitate the contraction of the targeted veins and lead to increased interaction between POL and the vein endothelium.

Regarding the current research, our study had enough sample size. There were many unpredictable and unable‐to‐standardize factors, such as the tap's angle, the syringas' temperature, and the warming of the agent. We obtained different FHTs as if we created the foam under the same condition. We excluded some preparation settings that limited our results. Another limitation of the study is to evaluate the stability of foam with only FHT. A bubble diameter examination could be beneficial, too. The decay process is another topic on sclerosing stability. The conditions can alter the foam stabilization by reducing surface tension, decreasing coalescence, Ostwalt ripening, and bubble collapsing; however, we did not explore this. Instead, assessing FHT with reproducibility became our priority.

In sum, the novel approach revealed that the temperature can mainly alter the FHT rather than the agent concentration or liquid–gas ratio. Thus, practitioners may achieve more stable foam at lower temperatures. The agent's concentration impacts FHT via the foam ionic charge and surface tension until CMC. Low temperature may aid in increasing POL's CMC by altering liquid viscosity. The effect of the temperature on the FHT may be increasing the CMC and may lead to elevating the sclerosant agent's upper concentration limit. Further research on determining the CMC of sclerosant agents would be more promising.

## 5 CONCLUSIONS

Physicians can create the foam under several conditions. However, foam stability needs improving approaches. Adverse effects in applications might decrease as FHT increases. Thus, experimental research on the strength can illuminate optimal production. Concentration and the ratio of liquid–air generally depend on the doctor's preferences and the diameter of the target vessel. However, low temperatures can mainly improve longevity. New studies to define the CMC of the POL with clinical and experimental research are promising.

## ETHICS STATEMENT

This study does not involve any human participants and was conducted entirely in vitro. Therefore, no ethical approval or informed consent was required.

## Data Availability

Data available on request from the authors.

## References

[1] Bai T , Jiang W , Chen Y , Yan F , Xu Z , Fan Y . Effect of multiple factors on foam stability in foam sclerotherapy. Sci Rep. 2018;8(1):15683. 10.1038/s41598-018-33992-w PMC6200734 30356108

[2] Shi X , Liu Y , Li D , Tursun M , Azmoun S , Liu S . The stability of physician‐compounded foam is influenced by the angle of connector. Ann Vasc Surg. 2023;99:217‐222. 10.1016/j.avsg.2023.08.011 37852364

[3] Bai T , Chen Y , Jiang W , Yan F , Fan Y . Studies on foam decay trend and influence of temperature jump on foam stability in sclerotherapy. Vasc Endovascular Surg. 2018;52(2):98‐106. 10.1177/1538574417741786 29173136

[4] Bai T , Liu Y , Jiang W , et al. A review of sclerosing foam stability in the treatment of varicose veins. Dermatol Surg. 2020;46(2):249‐257. 10.1097/DSS.0000000000002039 31569112

[5] Tessari L , Cavezzi A , Frullini A . Preliminary experience with a new sclerosing foam in the treatment of varicose veins. Dermatol Surg. 2001;27(1):58‐60. 11231246

[6] Zhang H‐S , Yang A‐J , Liu S . Identifying the most stable state of a foam sclerosant for foam sclerotherapy. Dermatol Surg. 2020;46(10):e66‐e70. 10.1097/DSS.0000000000002311 31895253

[7] Nastasa V , Samaras K , Ampatzidis C , et al. Properties of polidocanol foam in view of its use in sclerotherapy. Int J Pharm. 2015;478(2):588‐596. 10.1016/j.ijpharm.2014.11.056 25433198

[8] Valenzuela GC , Wong K , Connor DE , Behnia M , Parsi K . Foam sclerosants are more stable at lower temperatures. Eur J Vasc Endovasc. 2013;46(5):593‐599. 10.1016/j.ejvs.2013.08.012 24070851

[9] Tessari L . Nouvelle technique d'obtention de la scleromousee. Phlebologie. 2000;53:129.

[10] Cavezzi A , Parsi K . Complications of foam sclerotherapy. Phlebology. 2012;27(Suppl 1):46‐51. 10.1258/phleb.2012.012s09 22312067

[11] Yiannakopoulou E . Safety concerns for sclerotherapy of telangiectases, reticular and varicose veins. Pharmacology. 2016;98(1–2):62‐69. 10.1159/000445436 27104778

[12] Cavezzi A , Tessari L . Foam sclerotherapy techniques: different gases and methods of preparation, catheter versus direct injection. Phlebology. 2009;24(6):247‐251. 10.1258/phleb.2009.009061 19952380

[13] de Ávila Oliveira R , Riera R , Vasconcelos V , Baptista‐Silva JC . Injection sclerotherapy for varicose veins. Cochrane Database Syst Rev. 2021;12(12):Cd001732. 10.1002/14651858.CD001732.pub3 PMC8660237 34883526

[14] Erkin A , Kosemehmetoglu K , Diler MS , Koksal C . Evaluation of the minimum effective concentration of foam sclerosant in an ex‐vivo study. Eur J Vasc Endovasc. 2012;44(6):593‐597. 10.1016/j.ejvs.2012.09.021 23122185

[15] Goldman MP , Bergan JJ , Guex J‐J , Partsch H , Ramelet A‐A , eds. 7—mechanism of action of sclerotherapy. Sclerotherapy. 4th ed. Mosby; 2007:163‐188.

[16] Cameron E , Chen T , Connor DE , Behnia M , Parsi K . Sclerosant foam structure and stability is strongly influenced by liquid air fraction. Eur J Vasc Endovasc Surg. 2013;46(4):488‐494. 10.1016/j.ejvs.2013.07.013 23993276

[17] Lin‐Lin Y , Yi‐Fei W , Jian‐Yong D , Tao W . Influence of temperature on the stability of lauromacrogol foam for sclerotherapy. China J Oral Maxillofac Surg. 2017;15(3):198.

